# Low Intelligence Predicts Higher Risks of Coronary Artery Disease and Myocardial Infarction: Evidence From Mendelian Randomization Study

**DOI:** 10.3389/fgene.2022.756901

**Published:** 2022-02-07

**Authors:** Fangkun Yang, Teng Hu, Songzan Chen, Kai Wang, Zihao Qu, Hanbin Cui

**Affiliations:** ^1^ Department of Cardiology, Ningbo Hospital of Zhejiang University (Ningbo First Hospital), School of Medicine, Zhejiang University, Ningbo, China; ^2^ School of Medicine, Ningbo University, Ningbo, China; ^3^ School of Medicine, Zhejiang University, Hangzhou, China; ^4^ Cardiology Center, Ningbo First Hospital, Ningbo University, Ningbo, China

**Keywords:** intelligence, coronary artery disease, myocardial infarction, Mendelian randomization, causal association

## Abstract

**Background:** Low intelligence has been shown to be associated with a high risk of cardiovascular disease in observational studies. It remains unclear whether the association is causal. This study aimed to explore the causal association of intelligence with coronary artery disease (CAD) and myocardial infarction (MI).

**Methods:** A two-sample Mendelian randomization study was designed to infer the causality. A total of 121 single nucleotide polymorphisms were selected as a genetic instrumental variable for intelligence. Summary data on CAD (*n* = 184,305) and MI (*n* = 171,875) were obtained from the Coronary ARtery DIsease Genome-wide Replication and Meta-analysis (CARDIoGRAM) plus The Coronary Artery Disease (C4D) Genetics (CARDIoGRAMplusC4D) consortium and the FinnGen study. Inverse variance weighting method was used to calculate the effect estimates. Sensitivity analyses including other statistical models and leave-one-out analysis were conducted to verify the robustness of results. MR-Egger test was performed to assess the pleiotropy.

**Results:** Genetically predicted higher intelligence was significantly associated with lower risk of CAD (OR, .76; 95%CI, .69–.85; *p* = 1.5 × 10^–7^) and MI (OR, .78; 95%CI, .70–.87; *p* = 7.9 × 10^–6^). The results remained consistent in the majority of the sensitivity analyses and were repeated in the FinnGen datasets. MR-Egger test suggested no evidence of directional pleiotropy for the association with coronary artery disease (intercept = −.01, *p* = .19) and myocardial infarction (intercept = −.01, *p* = .06).

**Conclusion:** This Mendelian randomization analysis provided genetic evidence for the causal association between low intelligence and increased risks of CAD and MI.

## Introduction

Cardiovascular diseases (CVD) have represented a major cause of death and disability in the past few decades (Joseph et al., 2017). The global number of deaths associated with CVD has increased by 12.5% during the past 10 years (GBD 2015 Maternal Mortality Collaborators, 2016). In Europe, CVD cause more than 4 million deaths each year, accounting for 45% of all deaths (Townsend et al., 2016). The burden of CVD remains a great challenge, though great efforts have been made to manage this disease (GBD 2013 Mortality and Causes of Death Collaborators, 2015).

In addition to diagnosis and treatment, prevention strategies for CVD are also indispensable (Goff et al., 2014). Many risk factors have been found independently associated with CVD, such as age, sex, hyperlipidemia, hypertension, diabetes, smoking, and family history (Chiu et al., 2018; Madhavan et al., 2018). The risk of incidence of CVD could be effectively reduced by intervening on several modifiable factors among them. There are also other newly discovered risk factors. Several observational studies demonstrated that low intelligence is associated with a high risk of CVD (Roberts et al., 2013; Dobson et al., 2017). However, it is unclear whether this association is causal or spurious.

Randomized controlled trials (RCTs) are the most reliable methods to explore the direct association between the exposures and the outcomes. However, these trials are difficult to carry out due to ethics or others. In recent years, Mendelian randomization (MR) studies, considered an analogy of RCTs, have been increasingly used to ascertain the cause of diseases (Bennett and Holmes, 2017). MR studies use genetic variations as instrumental variables for the exposures, randomly allocated at conception (Sekula et al., 2016; Emdin et al., 2017). Therefore, MR studies are less prone to environmental confounders. Moreover, reverse causality is avoided considering that alleles were always allocated before the onset of the diseases (Sekula et al., 2016; Emdin et al., 2017). A recent regression analysis and MR study investigated the association between intelligence and coronary artery disease (CAD) risk (Li et al., 2021). However, the MR part was simple and not rigorous enough in the selection of the instrumental variable, outcome dataset, and statistical methods. Moreover, the role of physical activity, alcohol use, sleep traits, and psychological factor needs to be further investigated.

This study aims to resort to the MR study to provide consistent evidence for the causal association of genetically determined intelligence with the risk of CAD and myocardial infarction (MI).

## Materials and Methods

### Study Design

A two-sample MR study was designed to estimate the causal association between intelligence and the risk of CAD and MI. Three core assumptions for identifying the genetic instrumental variables are the basis of the MR analyses (Sekula et al., 2016; Emdin et al., 2017). First, the genetic instruments should be strongly associated with intelligence, generally at the genome-wide significant level (*p* < 5 × 10^–8^). Second, the instruments should be independent of the confounders. Third, the instruments should be only associated with the CAD and MI *via* intelligence.

### Construction of the Genetic Instrumental Variable

The exposure was genetically predicted intelligence. Genetic associations with intelligence were taken from the largest meta-analysis of the genome-wide association study (GWAS) of intelligence to date (*n* = 269,867) (Savage et al., 2018). That meta-analysis included 14 independent cohorts of European ancestry, adjusted for age, sex, and ancestry principal components. Although intelligence was assessed using different neurocognitive tests in each cohort, the cognitive test scores remained robust in multiple populations (Savage et al., 2018). In that study, 242 lead single-nucleotide polymorphisms (SNPs) were identified as significantly associated with intelligence at a genome-wide significant level (*p* < 5 × 10^–8^). The SNPs were further quality-controlled based on a minor allele frequency >1%. For palindromic SNPs, if the minor allele frequency is smaller than .42, then this SNP was regarded as inferrable. Any palindromic SNPs with minor allele frequency larger than .42 were regarded as not inferrable and would be removed. The pairwise-linkage disequilibrium of SNPs was tested using LD-Link (https://ldlink.nci.nih.gov/) based on the European 1,000 Genomes Project reference panel (*r* ^2^ < .001 and clump distance >10,000 kb). If SNPs were in linkage disequilibrium, the SNP with a greater *p*-value would be removed (Machiela and Chanock, 2015; Myers et al., 2020). Then, those 157 SNPs were looked up in PhenoScanner 2.0 (a database of human genotype-phenotype associations) manually (Staley et al., 2016). The SNPs associated with other traits that may influence the results at a genome-wide significance level (*p* < 5 × 10^–8^) were further removed. We found that 25 SNPs were associated with body mass index, height, weight, or waist circumference and 11 SNPs were associated with cholesterol level, blood pressure, diabetes, alcohol intake, or smoking (Supplementary Table 1). After excluding these 36 SNPs, the remaining 121 SNPs were finally selected as the instrumental variable of intelligence.

### Data Sources

The summary statistics for genetic associations with CAD and MI were acquired from Coronary ARtery DIsease Genome-wide Replication and Meta-analysis (CARDIoGRAM) plus The Coronary Artery Disease (C4D) Genetics (CARDIoGRAMplusC4D) consortium [*n* = 184,305, the majority (77%) were of European ancestry] (Nikpay et al., 2015). That study involved 60,801 CAD cases (∼70% were MI sub-phenotype) and 123,504 controls. The participants were phenotyped based on clinical diagnosis and medical records. The data was publicly available in CARDIoGRAMplusC4D consortium (http://cardiogramplusc4d.org/). The replication datasets were from the FinnGen study, which was launched in Finland in 2017, including genome and health data from about 500,000 Finnish participants (https://www.finngen.fi/en). We used the fifth release of the results of genome-wide association analysis on CAD, including 21,012 cases and 197,780 controls. The genetic associations for MI included 12,801 cases and 187,840 controls. Genetic associations with smoking and alcohol use were obtained from the GWAS and Sequencing Consortium of Alcohol and Nicotine (GSCAN) use (Liu et al., 2019). Genetic associations with physical activity were acquired from a GWAS including about 90,000 individuals of European ancestry (Doherty et al., 2018). Genetic associations with sleep duration and insomnia were from the Sleep Disorder Knowledge Portal (Dashti et al., 2019; Jansen et al., 2019). Genetic associations with depression were obtained from Psychiatric Genomics Consortium (PGC) (Howard et al., 2019). Studies contributing data to the outcome datasets had already received ethical approval from relevant institutional review boards. In the present study, we only made use of the summarized data from these studies. Hence, no additional ethics approval was required.

### Statistical Analyses

Two-sample MR analyses were used to estimate the causal associations of intelligence with the risk of CAD and MI. Specifically, we calculated the Wald ratio (quotient of the genetic association with outcome and the genetic association with the intelligence) and standard error for each SNP and then meta-analyzed them using the inverse variance weighting (IVW) method with fixed effect as our main MR effect estimates. In the sensitivity analyses, in order to test the robustness of the main results, the MR analyses with various statistical models, such as maximum likelihood, the IVW with multiplicative random effect (Bowden et al., 2017), penalized IVW, penalized robust IVW, simple median, weighted median (Bowden et al., 2016), and Mendelian Randomization Pleiotropy Residual Sum and Outlier (MR-PRESSO) (Verbanck et al., 2018), were conducted. The MR-Egger intercept test was used to assess the violation of the “no directional pleiotropy” assumption (Bowden et al., 2015). The visual inspection of scatter plots, funnel plots (Sterne et al., 2011), and leave-one-out plots were also performed to detect the potential horizontal pleiotropy (Bowden et al., 2017). Multivariable MR analysis was performed to investigate whether the association between intelligence and CAD/MI would be affected by potential confounders, including lifestyle factors [smoking (Liu et al., 2019), drinking (Liu et al., 2019), physical activity (Doherty et al., 2018), sleep duration (Dashti et al., 2019), insomnia (Jansen et al., 2019)], and psychological factor [depression (Howard et al., 2019)] (Burgess and Thompson, 2015; Sanderson et al., 2019). Specifically, we obtained summary-level data of the intelligence-related SNPs with confounding factors from corresponding genetic consortia. Then, the data was combined with the genetic associations between intelligence and outcomes for each SNP. The multivariable MR analysis allowed the genetic variants to be associated with all the risk factors in the statistical model (Burgess and Thompson, 2015). Causal estimates reflecting direct causal effects of the primary risk factor were provided, adjusted for the influence of a secondary risk factor or mediator. For power calculation, we used an online tool named mRnd (https://shiny.cnsgenomics.com/mRnd/) based on sample size, type-I error rate, proportion of cases, odds ratio of outcome per standard deviation of exposure, and proportion of variance explained by the included SNPs (Freeman et al., 2013). All the analyses needed to achieve the statistical power of at least 80%. A two-sided *p*-value of <.025 (=.05/2 outcomes) was defined as statistically significant. All the statistical analyses in the current study were implemented by the R software (version 3.6.3) together with the R package “MendelianRandomization” (https://github.com/cran/MendelianRandomization) and “MR-PRESSO” (https://github.com/rondolab/MR-PRESSO) (Yavorska and Burgess, 2017; Verbanck et al., 2018). In the MR analyses using the IVW method, we chose the fixed-effect, random-effect, penalized, or robust model. And for other analyses, default settings were used.

## Results

After excluding SNPs that might violate the three core assumptions, 121 SNPs were identified as a genetic instrument in our main analysis. The characteristics of these SNPs and their genetic associations with the intelligence and the outcome are shown in Supplementary Table 2.

**FIGURE 1:**
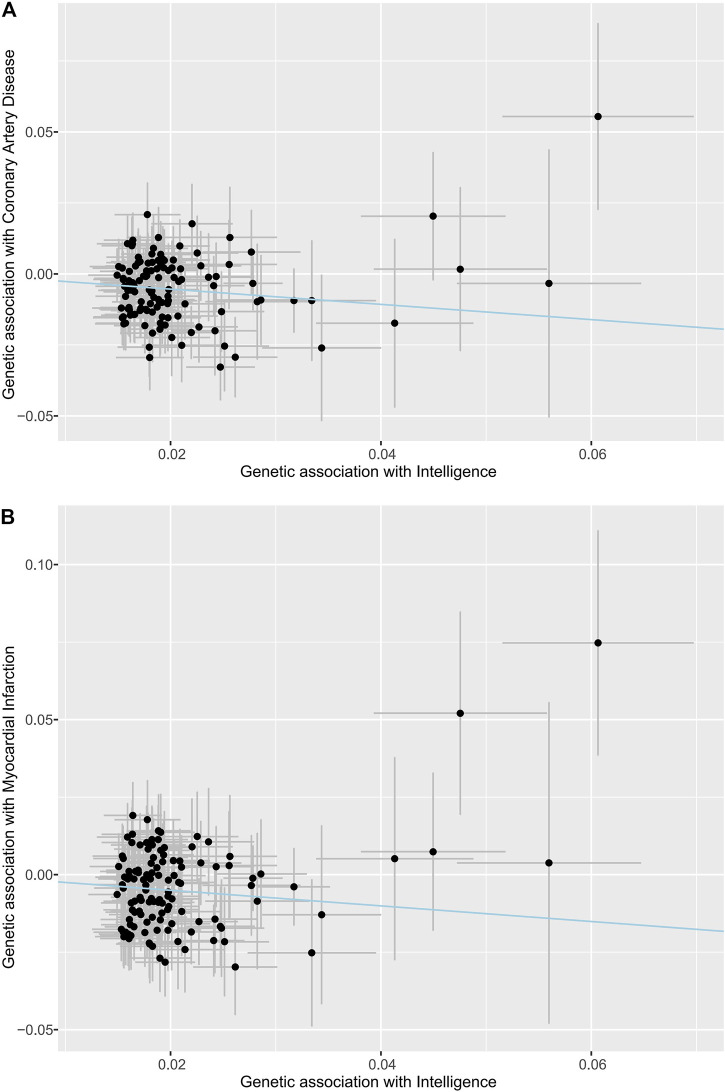
Associations of intelligence-related variants with outcomes. **(A)** Coronary artery disease. **(B)** Myocardial infarction. The dots indicate the causal effect of each SNP. The bars indicate the 95% confidence intervals. The blue line indicates the estimate of effect using the inverse-variance weighted method.

**FIGURE 2:**
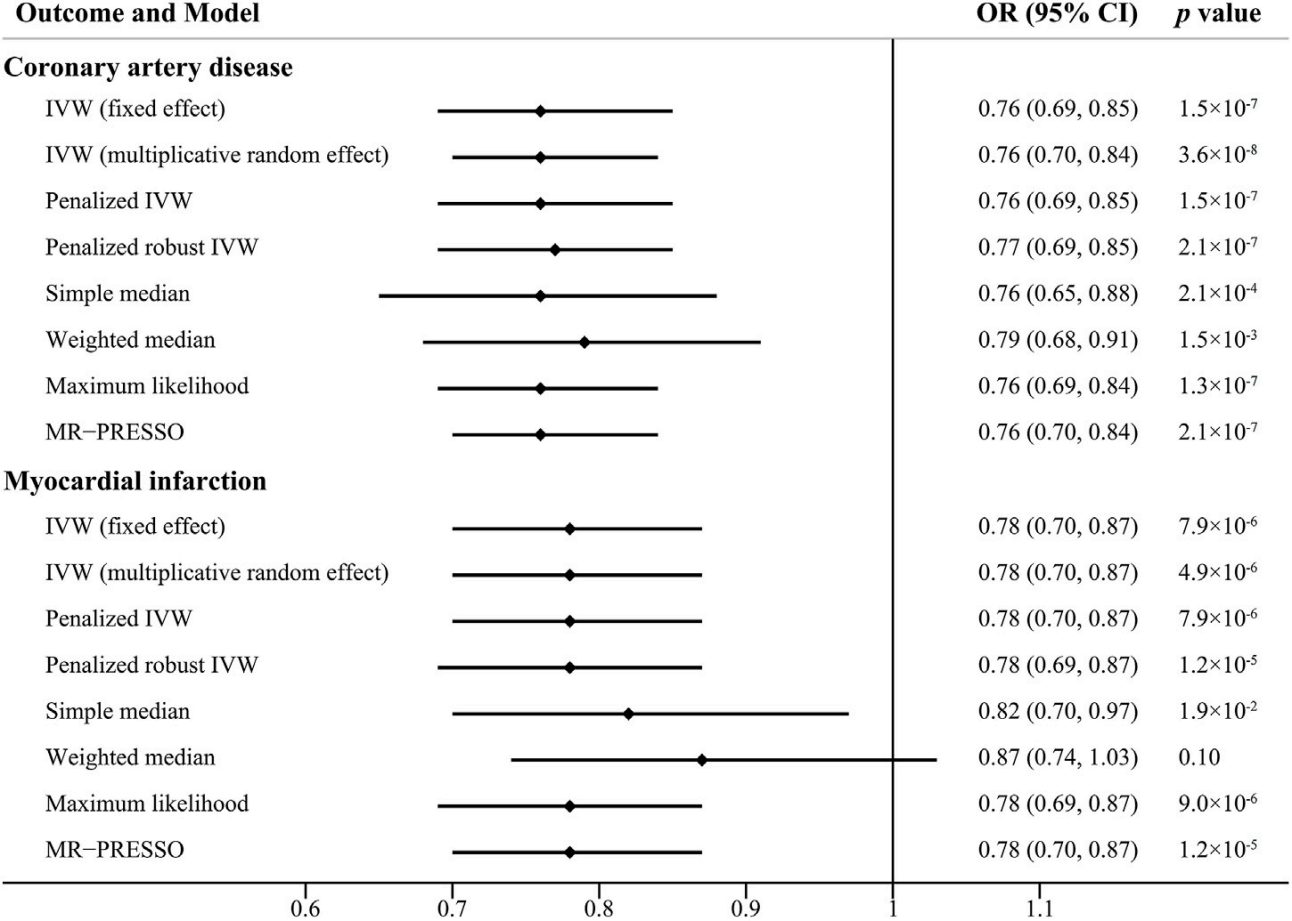
Causal effect estimates of genetically predicted intelligence on coronary artery disease and myocardial infarction using different statistical models. OR, odds ratio; CI, confidence interval.

The scatter plots of the associations between the genetically predicted intelligence and CAD/MI are displayed in Figure 1. The associations between the genetically predicted intelligence and CAD/MI are shown in Figure 2. The fixed-effect IVW method showed that higher genetically predicted intelligence was significantly associated with lower risks of CAD (OR .76 per SD increase; 95%CI, .69–.85; *p* = 1.5 × 10^–7^) and MI (OR .78 per SD increase; 95%CI, .70–.87; *p* = 7.9 × 10^–6^). Similar results were observed using the maximum likelihood, the multiplicative random effect IVW, penalized IVW, penalized robust IVW, simple median, weighted median (for CAD), and MR-PRESSO method. However, the associations were not evident using the weighted median (for MI) and MR-Egger test. The main results were repeated based on genetic data for CAD and MI from the FinnGen study, indicating the robustness and consistency of the main results (Table 1; Supplementary Table 3).

**TABLE 1:** The associations between intelligence and coronary artery disease/myocardial infarction using genetic data from the FinnGen study.

Outcome	Statistical model	OR	95% CI	*p*-value
CAD	IVW (random effects)	.82	(.70, .95)	6.7E-03
	IVW (fixed effects)	.82	(.71, .94)	4.6E-03
	Weighted median	.91	(.74, 1.12)	.36
	MR-Egger	1.47	(.71, 3.03)	.30
	Maximum likelihood	.81	(.70, .94)	4.5E-03
	MR-PRESSO	.82	(.70, .95)	7.7E-03
MI	IVW (random effects)	.76	(.64, .89)	9.2E-04
	IVW (fixed effects)	.76	(.64, .90)	1.2E-03
	Weighted median	.80	(.63, 1.01)	6.3E-02
	MR-Egger	1.06	(.46, 2.46)	.89
	Maximum likelihood	.75	(.63, .89)	1.2E-03
	MR-PRESSO	.76	(.64, .89)	1.2E-03

CAD, coronary artery disease; MI, myocardial infarction; IVW, inverse-variance weighted; MR-PRESSO, mendelian randomization pleiotropy residual sum and outlier; OR, odds ratio; CI, confidence interval.

**FIGURE 3:**
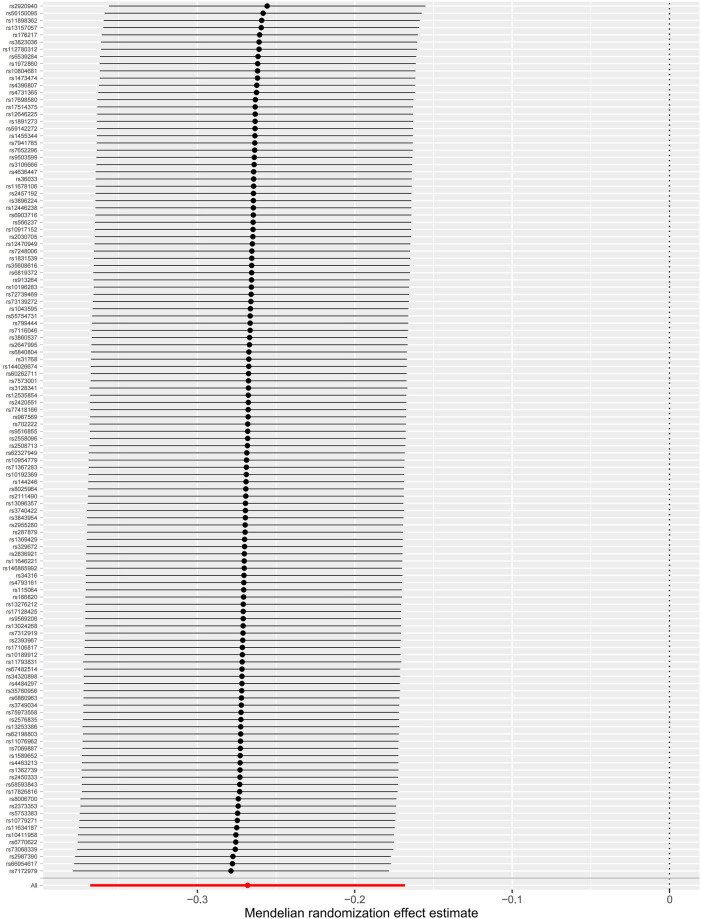
Leave-one-out analyses of the associations between intelligence and coronary artery disease. The dots indicate the causal effect using the inverse-variance weighted method when the SNP is removed. The bars indicate a 95% confidence interval.

The MR-Egger intercept test is shown in Table 2, which did not provide strong evidence of potential directional pleiotropy for the associations between the genetically predicted intelligence and CAD (intercept = −.01, *p* = .19) and MI (intercept = −.01, *p* = .06). Funnel plots were symmetric distribution, indicating no obvious potential pleiotropic effects (Supplementary Figure 1). The results of the leave-one-out analysis suggested that the associations between the genetically predicted intelligence and CAD/MI were stable and not drastically driven by individual SNP (Figure 3; Supplementary Figure 2). The association pattern remained after adjusting for most of the potential confounding traits (Table 3). The MR estimates were slightly attenuated after adjusting for smoking and sleep duration. However, limited evidence was found for the mediating effect of smoking and sleep duration between intelligence and CAD/MI. The MR analyses have 98% and 90% statistical power at the type I error rate of .05 for association with CAD and MI, respectively (Supplementary Table 4).

**TABLE 2:** MR-Egger tests of intelligence with CAD and MI.

Outcome	MR-Egger	Estimate	LCI	UCI	*p*-value
CAD	Slope	.06	−.45	.56	.82
	Intercept	−.01	−.02	.0033	.20
MI	Slope	.27	−.29	.83	.34
	Intercept	−.01	−.02	.0005	.06

LCI, lower confidence interval; UCI, upper confidence interval; CAD, coronary artery disease; MI, myocardial infarction.

**TABLE 3:** Multivariable Mendelian randomization analyses of genetically determined intelligence and the risk of coronary artery disease and myocardial infarction adjusting for potential confounding traits.

Outcome	Adjusting for	OR	95% CI	*p*-value
CAD	Smoking	.76	(.69, .85)	5.5E-07
	Alcohol use	.76	(.69, .84)	1.3E-07
	Physical activity	.76	(.68, .84)	9.4E-08
	Insomnia	.76	(.69, .84)	1.3E-07
	Sleep duration	.77	(.70, .85)	4.9E-07
	Depression	.77	(.69, .85)	2.4E-07
MI	Smoking	.79	(.70, .88)	5.4E-05
	Alcohol use	.77	(.69, .87)	7.6E-06
	Physical activity	.77	(.69, .86)	7.6E-06
	Insomnia	.77	(.69, .87)	7.2E-06
	Sleep duration	.78	(.70, .87)	1.4E-05
	Depression	.78	(.70, .87)	1.4E-05

CAD, coronary artery disease; MI, myocardial infarction; OR, odds ratio; CI, confidence interval.

## Discussion

We conducted a two-sample MR study to explore the causal effects of intelligence on CAD and MI. We found that the higher genetically predicted intelligence was significantly associated with the lower risk of CAD and MI. In addition, the results remained consistent in the majority of the sensitivity analyses with different statistical models and leave-one-out analyses.

CVD represents a leading cause of illness and disability associated with high morbidity and mortality (Benjamin et al., 2018). Except for the well-established risk factors, such as age, sex, and hypertension, intelligence has been newly discovered as an intriguing risk factor (Dobson et al., 2017). In the past few decades, observational epidemiological studies have accumulated evidence for an inverse association between intelligence and the risk of CVD. A prospective cohort study in Scotland with 938 participants and a 25-year follow-up showed that childhood intelligence quotient (IQ) was significantly inversely related to CVD events in individuals aged up to 65 (Hart et al., 2004). The Newcastle Thousand Families study with 412 members and a 40-year follow-up suggested that individuals with higher childhood intelligence had a lower risk of atherosclerosis in middle age (Roberts et al., 2013). In a meta-analysis of five longitudinal studies with 17,256 participants, each standard deviation decrease in childhood IQ was associated with an increase of 16% in the risk of CVD (Dobson et al., 2017). Moreover, a prospective cohort study of 49,321 Swedish males and another cohort study of 4,316 Vietnam males demonstrated that lower IQ scores in early adulthood were associated with an increased risk of coronary heart disease (CHD) and acute myocardial infarction (AMI) (Hemmingsson et al., 2007; Batty et al., 2008). However, these studies fail to distinguish between the causal and spurious associations because of the unmeasured confounding and reverse causality. The present study can largely overcome these shortcomings and provide a reliable causal inference. Our study, together with previous evidence, suggested that intelligence was causally associated with the risks of CAD and MI.

Though the associations between the low premorbid intelligence and the increased risk of CVD and the high rate of later mortality have been explored, the exact mechanism remains unclear. Several plausible hypotheses have been proposed. First, socioeconomic factor was put forward to explain the association between intelligence and CAD risk. Individuals with low intelligence were less prone to educational success and well-remunerated employment, which provided protection against CAD (Batty et al., 2009). Second, the effect of intelligence could be mediated *via* health literacy. Individuals with low intelligence were less likely aware of their health conditions and even had more difficulties understanding health messages (Dobson et al., 2017). They rarely knew how to prevent the diseases or take medicine properly. It was demonstrated that higher intelligence was associated with improved disease prevention or better health behaviors, including quitting smoking, having a prudent diet, and persisting with moderate physical activity (Sörberg Wallin et al., 2015). However, the present MR study found limited evidence for the mediating effect of smoking, physical activity, and sleep duration between intelligence and CAD/MI. Third, these associations could also be partly explained by the congenital or childhood health damage in both intelligence and physiological functions, which eventually increased CAD risk in later life (Dobson et al., 2017). Future investigations were warranted to elucidate the exact mechanism by which the low intelligence was causally associated with the increased CAD risk.

The strength of this study is the design of the two-sample MR study. MR analysis is a novel technique that uses genetic variants as instrumental variables to estimate the causal effect of exposure on the outcomes (Sekula et al., 2016). The genetic variants are not associated with other confounding factors because of the random allocation at the conception, greatly reducing the potential bias (Sekula et al., 2016; Emdin et al., 2017). MR analysis can also avoid reverse causation because genotyping is always earlier than phenotyping (Sekula et al., 2016; Emdin et al., 2017). MR analysis represents a reliable method to infer the causal associations, even described as the best alternative to RCTs (Nitsch et al., 2006). In addition, we investigated the causal association between intelligence and CAD and MI based on a large-scale cohort, which could improve the effectiveness of the statistical test.

There are several limitations to our study. First, the potential pleiotropy cannot be completely ruled out, which may lead to biased causal estimates. However, the MR-Egger intercept test suggested no potential directional pleiotropy, and MR-PRESSO found no evidence of horizontal pleiotropic outliers. The result was almost consistent in the sensitivity analysis except for the MR-Egger method. On the one hand, it could be explained by a potential violation of the Instrument Strength Independent of Direct Effect (InSIDE) assumption. This assumption was unlikely completely satisfied, to which the MR-Egger method was sensitive. On the other hand, MR-Egger regression was very conservative compared to other methods, especially when no violation of the horizontal pleiotropy assumption was evident. Second, we did not explore the associations between the genetic instrumental variables and the observed confounders, such as body mass index and cholesterol level. Nevertheless, we had excluded the SNPs related to potential confounders by looking up the SNPs in PhenoScanner. Third, the phenotype of the intelligence varied among the 14 independent cohorts included in the GWAS, but the test scores remained robust in multiple populations. This result needed to be verified when a uniform and precise phenotype of the intelligence was available in GWAS studies. Moreover, the results of the current study were based on samples from individuals of European ancestry, and the effect of low intelligence on the risk of CAD/MI needed to be further investigated in other racial and ethnic groups. Finally, we only revealed the causal association between intelligence and CAD and MI from a genetic perspective without involving other environmental factors.

## Conclusion

Our two-sample MR study provides genetic evidence for the causal association between low intelligence and the increased risk of CAD and MI. Early recognition coupled with appropriate care of individuals with low intelligence may have significant clinical and public health implications. Further studies are warranted to verify our findings and reveal the potential mechanism.

## Data Availability

The datasets presented in this study can be found in online repositories. The names of the repository/repositories and accession number(s) can be found in the article/Supplementary Material.
