# Supplementary material for: Low Intelligence Predicts Higher Risks of Coronary Artery Disease and Myocardial Infarction: Evidence From Mendelian Randomization Study
Source: Front Genet. 2022 Feb 7;13:756901. doi: 10.3389/fgene.2022.756901 (PMC8859249; doi:10.3389/fgene.2022.756901)
Supplement: Supplementary file 1 [file DataSheet1.PDF]

Supplemental Material

Supplementary Table 1. Association between 36 SNPs and other traits at genome-wide significance level ($p<5\times10^{-8}$).

SNP	EA	OA	EAF	Other traits
rs7546297	a	g	0.378	Height; Whole body fat-free mass
rs2842188	t	c	0.352	Height
rs6668048	t	c	0.472	Alcohol usually taken with meals
rs1144593	a	g	0.703	LDL cholesterol; Total cholesterol
rs2678210	t	c	0.714	Body mass index; Weight
rs10189857	a	g	0.563	Height
rs2309812	t	c	0.358	Body mass index; Waist circumference
rs4687625	t	c	0.444	Body mass index
rs11720523	a	c	0.410	Alcohol intake frequency
rs9853960	a	g	0.329	Body mass index

rs34811474	a	g	0.198	Body mass index
rs4459994	a	c	0.224	Birth weight
rs1812587	t	g	0.483	Body mass index
rs76160968	a	g	0.962	Diabetes diagnosed by doctor
rs35433030	a	g	0.083	Diastolic blood pressure
rs1280049	a	c	0.475	Height
rs13212044	t	g	0.231	High blood pressure
rs4725065	a	g	0.518	Height
rs1106761	a	g	0.365	Body mass index
rs28620532	a	g	0.644	Height
rs11605348	a	g	0.342	Body mass index
rs2885208	t	c	0.808	Past tobacco smoking

rs1962047	a	g	0.362	Height
rs1727307	a	g	0.289	Height
rs1007934	a	g	0.380	Body mass index
rs34172651	t	c	0.679	Weight; Systolic blood pressure; Waist circumference
rs8054299	c	g	0.682	Height; Weight
rs9888986	a	g	0.116	High density lipoprotein
rs2285640	a	g	0.544	Body mass index
rs17698176	t	g	0.802	Height; Systolic blood pressure
rs11079849	t	c	0.313	Body mass index
rs76608582	a	c	0.039	Past tobacco smoking
rs2072490	t	c	0.509	Body mass index
rs889169	a	g	0.601	Body mass index

rs78084033	a	c	0.866	Weight; Hip circumference
rs6019535	a	g	0.305	Height

SNP, single-nucleotide polymorphism; EA, effect allele; OA, other allele; EAF, frequency of effect allele.

Supplementary Table 2. The characteristics of 121 SNPs and their genetical associations with the intelligence and the outcome.

SNP	Chr	Pos	EA	OA	EAF	Intelligence		CAD		MI	
						Beta	SE	Beta	SE	Beta	SE
rs10917152	1	22425642	t	c	0.131	0.02421334	0.00404905	-0.020027	0.015726	-0.0143478	0.0174581
rs4636447	1	41835633	a	g	0.606	0.01765726	0.00281435	-0.012485	0.0096212	-0.0034009	0.0105967
rs1831539	1	59560337	t	c	0.543	-0.0172035	0.00276228	0.00984	0.0093605	0.0083561	0.0103598
rs2420551	1	69438777	a	t	0.887	-0.0285957	0.00434387	0.009178	0.0158499	-0.0002285	0.0176047
rs3128341	1	72749848	t	c	0.198	-0.031727	0.00341739	0.009358	0.0113201	0.00392264	0.0125239
rs1473474	1	98588885	t	c	0.472	0.01555762	0.00273757	-0.017575	0.0092822	-0.0199848	0.0102451
rs112780312	1	153797015	a	g	0.284	-0.0182838	0.00309947	0.02083	0.0102317	0.0231358	0.0112738
rs34320898	1	171759139	c	g	0.184	0.02286931	0.00384423	0.002826	0.0122988	0.00374709	0.0135811

rs10779271	1	216832989	a	g	0.681	0.01637512	0.00292517	0.010584	0.009921	0.0129969	0.0110092
rs12470949	2	23934816	t	c	0.284	-0.0171675	0.00302085	0.012079	0.0104913	0.0128812	0.0116917
rs967569	2	41616346	t	c	0.674	-0.0179757	0.00292716	0.005656	0.0101936	0.00882839	0.011147
rs2955280	2	44116836	t	c	0.528	-0.0149147	0.00273413	0.000449	0.0094832	0.00640913	0.0105052
rs58593843	2	60499463	a	g	0.097	-0.0276826	0.00465176	-0.007701	0.0148462	0.00345561	0.0162236
rs2576835	2	64306984	a	g	0.241	-0.0194098	0.00320559	-0.004767	0.0109681	-0.0087626	0.0117414
rs11898362	2	73565293	a	g	0.303	-0.0179807	0.00300781	0.025778	0.0104869	0.0220792	0.0112595
rs11678106	2	82444107	t	c	0.497	0.01608594	0.00274504	-0.012624	0.0095364	-0.0144739	0.0102527
rs60262711	2	117647820	t	c	0.385	0.01594918	0.00282536	-0.005814	0.0099905	-0.0013797	0.010765
rs2558096	2	137531830	t	g	0.420	-0.0156318	0.00277356	0.004353	0.0095951	0.00082573	0.0103324
rs10189912	2	144162609	a	g	0.646	-0.0193366	0.00285285	-0.002148	0.0106118	-0.006386	0.0113718
rs3106666	2	155547853	a	g	0.411	-0.0165882	0.00278	0.014258	0.0101035	0.0120965	0.0110424

rs10192369	2	161380888	a	g	0.513	-0.0160526	0.00274403	0.002365	0.0098721	-0.0010273	0.0106496
rs3749034	2	171673475	a	g	0.218	-0.0192573	0.00332194	-0.005309	0.0115145	-0.0041589	0.012419
rs10196283	2	180715335	a	c	0.842	-0.0207086	0.00375974	0.014833	0.0143486	0.021586	0.0153748
rs62198803	2	186208380	a	g	0.236	0.01906514	0.00322537	0.006846	0.0117025	0.0136394	0.012455
rs7573001	2	198929896	c	g	0.380	-0.0162512	0.00285961	0.005685	0.0098982	0.00911594	0.0105342
rs1455344	2	199516059	a	g	0.444	-0.0161757	0.00276933	0.014552	0.0096136	0.0161517	0.0104736
rs13024268	2	232483518	a	g	0.386	-0.0166607	0.00287898	-0.002779	0.0105814	-0.0010439	0.0114243
rs73139272	3	16858468	t	g	0.132	-0.0248376	0.00405711	0.013254	0.0141585	0.0172556	0.0157291
rs1589652	3	35538452	a	g	0.443	0.01709375	0.00275928	0.004332	0.0092258	0.0095472	0.0102566
rs13096357	3	48675856	a	c	0.114	0.02361356	0.00431771	-0.001152	0.0154816	0.0106026	0.0173413
rs6770622	3	85171415	a	g	0.042	-0.0449613	0.00686431	-0.020312	0.0225946	-0.0074075	0.0255079
rs7652296	3	89587262	a	g	0.610	0.01653311	0.00279938	-0.014294	0.0097396	-0.0168234	0.0108447

rs3860537	3	108103433	t	c	0.210	0.01885374	0.00340197	-0.009038	0.012364	-0.0089338	0.0136177
rs59142272	3	139708741	a	g	0.169	0.02269562	0.00368495	-0.018636	0.012565	-0.0151543	0.0139874
rs10804681	3	141660675	a	t	0.154	0.02105457	0.00379704	-0.025138	0.0129865	-0.0119256	0.0143488
rs12646225	4	696848	t	c	0.120	0.0251275	0.00421532	-0.025419	0.0159324	-0.0216693	0.0175854
rs4484297	4	16332382	c	g	0.251	0.01826667	0.00316032	0.004049	0.0109377	0.00956305	0.0121438
rs144246	4	17259908	a	g	0.370	0.01549106	0.00283979	-0.001943	0.0096027	0.00519834	0.0105641
rs67482514	4	65785448	c	g	0.756	-0.0178589	0.00322946	-0.003777	0.0106651	-0.0082245	0.0118214
rs6819372	4	67970101	a	g	0.493	-0.0197961	0.00272861	0.009433	0.0091247	0.00180493	0.0101729
rs1972860	4	94579640	a	g	0.321	-0.0175561	0.00293041	0.018204	0.0098058	0.018667	0.0110736
rs62327949	4	103216782	a	g	0.655	-0.0162424	0.00293821	0.00324	0.0099373	0.0196427	0.0110569
rs6840804	4	147769212	a	g	0.696	-0.0165876	0.0029663	0.006294	0.00977	0.00859229	0.0109359
rs17826816	5	7519298	a	g	0.774	0.01837523	0.00325687	0.009005	0.0115667	0.00553009	0.0126474

rs75973558	5	26880925	a	g	0.885	0.02563562	0.00446536	0.012872	0.0178399	0.00590476	0.019794
rs13157057	5	57657757	t	g	0.240	0.01802374	0.00321336	-0.029439	0.0115623	-0.0223703	0.0126739
rs36033	5	60960727	t	c	0.582	0.01596854	0.0027878	-0.012292	0.0092975	-0.0192049	0.0102754
rs34316	5	88015545	a	c	0.435	0.02104905	0.00276743	-0.002004	0.0094267	0.00240018	0.0103318
rs166820	5	89353210	a	g	0.174	0.02433407	0.00359918	-0.000898	0.0119284	0.00257907	0.0130753
rs4463213	5	139545748	a	g	0.521	0.01906548	0.00273223	0.003055	0.0092985	0.0078211	0.0103013
rs31768	5	165498279	a	t	0.287	0.01817702	0.00305393	-0.00673	0.0105	-0.0077046	0.0117222
rs6860963	5	169239708	t	c	0.190	0.02026246	0.00347615	0.004886	0.0119034	0.00455162	0.0131337
rs2450333	5	176899546	a	g	0.495	-0.0188286	0.00279897	-0.004781	0.0100024	-0.0113045	0.0110386
rs9503599	6	3451048	t	c	0.563	-0.0171108	0.00278496	0.012908	0.0093561	0.00730874	0.0103516
rs566237	6	11543342	a	g	0.683	-0.0187161	0.0029354	0.011716	0.0096364	0.0179396	0.0106554
rs6903716	6	21956404	a	g	0.700	0.01775805	0.00297505	-0.012844	0.0101274	-0.0153706	0.0113069

rs77418166	6	98204498	t	c	0.092	0.02824364	0.00493684	-0.009765	0.0204188	-0.008542	0.0219905
rs3823036	6	99284532	t	c	0.681	-0.0189851	0.00292934	0.019438	0.0098851	0.0268994	0.01094
rs287879	6	157143339	a	g	0.731	-0.0188666	0.00307475	0.001336	0.010853	0.00022634	0.0118597
rs115064	7	24177191	t	c	0.612	0.01609617	0.00281402	0.000908	0.009468	-0.0005712	0.0105404
rs799444	7	44769190	t	c	0.451	0.01841498	0.00275921	-0.008252	0.0098645	-0.0087082	0.0110113
rs2030705	7	69713466	a	t	0.783	0.01825666	0.00330916	-0.013468	0.0109997	-0.01374	0.0123581
rs56150095	7	71759069	a	c	0.532	-0.0219682	0.00274706	0.02064	0.0093083	0.0184869	0.0103702
rs12535854	7	105063372	c	g	0.336	-0.0182259	0.00295299	0.005953	0.0103913	-0.0006064	0.0116706
rs4731365	7	127082497	a	g	0.393	-0.0192143	0.00279156	0.015211	0.0094495	0.00919797	0.0106832
rs1043595	7	128410012	a	g	0.261	0.01895674	0.00312251	-0.010082	0.0117448	-0.008079	0.0133192
rs1362739	7	133430934	a	c	0.469	0.02094523	0.00273365	0.00183	0.0092431	-0.002826	0.0103829
rs13253386	8	14002020	t	g	0.540	-0.0201329	0.00274814	-0.002131	0.0096027	0.00781934	0.0106395

rs10954779	8	31019597	t	c	0.556	-0.0163774	0.00276179	0.00306	0.0092736	0.0113514	0.0102687
rs13276212	8	66440593	t	g	0.483	0.01507074	0.00275466	0.002601	0.0097678	0.0026153	0.0107003
rs2920940	8	93180965	t	c	0.231	-0.0247422	0.00325171	0.032808	0.0117079	0.0166746	0.0127654
rs2111490	8	104561723	a	g	0.465	0.01549091	0.00275296	-0.001563	0.0095951	-0.0043263	0.0107049
rs11793831	9	23362311	t	g	0.408	0.02783379	0.00280441	-0.003313	0.009506	-0.0011453	0.0105701
rs702222	9	23805569	t	c	0.356	-0.0198308	0.00287195	0.005502	0.0094571	0.0102438	0.0105593
rs913264	9	131944138	t	c	0.287	0.01972454	0.0030257	-0.010503	0.0103425	-0.0112587	0.0116815
rs2987390	9	134710601	c	g	0.733	-0.0178028	0.00312274	-0.020877	0.0113451	-0.0177127	0.0127751
rs7069887	10	29569272	a	c	0.852	0.02253156	0.00389752	0.007382	0.0131277	0.0122682	0.0144908
rs2393967	10	65133156	a	c	0.691	-0.0187089	0.00296262	-0.001743	0.0103783	-0.0022412	0.0114552
rs1891273	10	93442379	t	c	0.489	0.01541437	0.0027864	-0.015409	0.0097461	-0.0032153	0.0108693
rs3740422	10	103565960	c	g	0.326	-0.0241019	0.00291156	0.004136	0.0104489	0.0212771	0.0115822

rs3896224	10	106467853	a	g	0.554	-0.0153148	0.00277191	0.012017	0.0092822	0.0176265	0.0102709
rs35608616	10	125429549	a	g	0.331	-0.0180894	0.00293706	0.010471	0.0101002	0.00135748	0.0111335
rs7941785	11	63861317	a	g	0.369	0.01551211	0.00284105	-0.01512	0.0098276	-0.0185765	0.0109933
rs2373353	11	79162622	a	g	0.637	-0.0163232	0.0028865	-0.009928	0.0101154	-0.0103682	0.0113366
rs2508713	11	95547927	a	t	0.362	0.0165308	0.00284083	-0.004168	0.0100687	-0.0013525	0.011203
rs7116046	11	105827909	t	c	0.371	0.0157066	0.00284231	-0.007862	0.0095897	-0.0008754	0.0106452
rs17128425	11	123983349	a	t	0.101	0.02555671	0.00454422	0.003341	0.0157206	0.00293753	0.0174621
rs329672	11	133799132	t	c	0.628	0.01743047	0.00285324	-0.000735	0.0097309	-0.001619	0.0108237
rs55754731	12	15532891	t	c	0.831	0.0213686	0.00367474	-0.010528	0.0126269	-0.0241785	0.013844
rs146865992	12	49385699	t	c	0.029	0.04754264	0.00822679	0.001695	0.0288787	0.0520784	0.032759
rs6539284	12	79592680	t	c	0.613	-0.0194809	0.00282741	0.01805	0.0099438	0.0282471	0.0110521
rs7312919	12	92960905	c	g	0.663	0.01814561	0.00291496	0.001112	0.0099112	0.00362202	0.0109853

rs9569206	13	55703218	a	g	0.629	-0.0154126	0.00282437	-0.002008	0.0094517	-0.0059767	0.0104723
rs3843954	13	58548511	c	g	0.274	-0.0207576	0.00334261	0.002645	0.0105999	0.0025193	0.0117342
rs9516855	13	97847992	a	g	0.947	0.03342678	0.00609644	-0.009388	0.0212411	-0.0252302	0.0237291
rs17514375	13	106593392	t	c	0.832	0.02010446	0.00364806	-0.02239	0.013585	-0.0158517	0.0150245
rs8006700	14	27162904	a	t	0.679	-0.0182269	0.00293038	-0.007037	0.0098471	-0.0113064	0.010947
rs176217	14	29600359	t	c	0.864	0.02614095	0.00398916	-0.029324	0.0141325	-0.0297959	0.015475
rs35760956	14	41134168	a	g	0.601	0.01979753	0.00282338	0.001196	0.0095495	-0.0056092	0.0106473
rs17106817	14	69716957	t	c	0.707	0.01691111	0.0030247	0.003981	0.0107107	0.0014299	0.0119238
rs17698580	14	98546911	t	c	0.756	0.01903298	0.00317693	-0.017292	0.0110648	-0.0145561	0.0122534
rs11634187	15	40722781	t	g	0.850	0.02203237	0.00385721	0.017662	0.0140477	0.00899616	0.0155594
rs7172979	15	51817198	t	g	0.024	0.0606345	0.00908382	0.055433	0.0328806	0.0747458	0.0363118
rs72739469	15	65738080	t	c	0.931	-0.0343573	0.00564809	0.026039	0.0257796	0.0129311	0.028832

rs8025964	15	82521770	a	g	0.465	0.01703061	0.00274865	-0.00241	0.0092562	-0.0118036	0.0102863
rs1369429	15	88430934	t	c	0.348	0.01763256	0.00289628	-0.000915	0.0097135	-0.0049514	0.0107983
rs11076962	16	5811367	t	c	0.717	0.01693623	0.00304171	0.005901	0.0101448	0.00063957	0.011318
rs11646221	16	7666088	t	g	0.556	0.01773538	0.00277159	-0.000686	0.0094017	-4.105E-05	0.0105849
rs2457192	16	12197441	a	c	0.723	-0.019751	0.00313111	0.01542	0.0118176	0.017921	0.0130915
rs2647995	16	51577196	t	c	0.705	-0.0197491	0.00304395	0.007842	0.0104402	0.00699174	0.0115705
rs12446238	16	62075138	a	g	0.459	0.01605412	0.00274242	-0.011826	0.0092768	-0.0206203	0.0103054
rs4793161	17	42919009	a	g	0.232	-0.0177187	0.00324995	-0.001072	0.0107324	-0.010339	0.0119079
rs66954617	17	56999427	a	g	0.374	-0.0208823	0.0028338	-0.00982	0.0093865	-0.0044057	0.0103976
rs71367283	18	50052084	a	c	0.972	0.05597403	0.00874594	-0.003343	0.0471879	0.00378044	0.0518644
rs10411958	19	13113641	t	c	0.472	0.01640856	0.00275867	0.011897	0.00972	0.0191087	0.0107523
rs7248006	19	31929180	t	c	0.388	-0.019175	0.00282027	0.010109	0.009531	0.0124007	0.0105086

rs144026674	19	39583980	t	c	0.038	0.04130659	0.0074682	-0.017357	0.0297249	0.00512416	0.0327433
rs73068339	19	59090263	c	g	0.283	0.01885817	0.00304557	0.012856	0.0106531	0.0141346	0.0118061
rs2836921	21	40516070	a	g	0.312	0.02034647	0.00296293	-0.001259	0.0104772	-0.0003161	0.0115593
rs5753383	22	31304201	a	g	0.321	0.01591002	0.00291873	0.01062	0.0099058	0.012112	0.0109712
rs4396807	22	38138379	c	g	0.361	-0.0157319	0.00285515	0.017338	0.0095984	0.0185962	0.0106245

SNP, single-nucleotide polymorphism; Chr, chromosome; Pos, position; EA, effect allele; OA, other allele; EAF, frequency of effect allele; SE, standard error; CAD, coronary artery disease; MI, myocardial infarction.

Supplementary Table 3. Summary statistics for coronary artery disease and myocardial infarction in the FinnGen study.

SNP	EA	Intelligence		CAD		MI	
		beta	se	beta	se	beta	se
rs10189912	A	-0.0193	0.0029	0.0007	0.0137	0.0165	0.0165
rs10192369	A	-0.0161	0.0027	0.0265	0.0129	0.0249	0.0156
rs10196283	A	-0.0207	0.0038	0.0557	0.0187	0.0459	0.0225
rs10411958	T	0.0164	0.0028	0.0078	0.0130	0.0027	0.0156
rs1043595	A	0.0190	0.0031	-0.0210	0.0157	-0.0178	0.0189
rs10779271	A	0.0164	0.0029	0.0075	0.0138	0.0037	0.0166
rs10804681	A	0.0211	0.0038	-0.0231	0.0170	-0.0235	0.0206
rs10917152	T	0.0242	0.0040	0.0271	0.0230	0.0390	0.0277
rs10954779	T	-0.0164	0.0028	0.0132	0.0134	0.0281	0.0161
rs11076962	T	0.0169	0.0030	0.0026	0.0150	-0.0020	0.0181
rs115064	T	0.0161	0.0028	0.0051	0.0139	0.0156	0.0168
rs11634187	T	0.0220	0.0039	-0.0199	0.0184	-0.0143	0.0221
rs11646221	T	0.0177	0.0028	0.0010	0.0132	0.0087	0.0159
rs11678106	T	0.0161	0.0027	-0.0024	0.0129	0.0046	0.0156
rs11793831	T	0.0278	0.0028	-0.0028	0.0130	0.0056	0.0157
rs11898362	A	-0.0180	0.0030	0.0188	0.0145	0.0107	0.0175
rs12446238	A	0.0161	0.0027	-0.0121	0.0130	-0.0039	0.0156
rs12470949	T	-0.0172	0.0030	-0.0062	0.0138	0.0078	0.0167
rs12535854	C	-0.0182	0.0030	-0.0064	0.0142	-0.0067	0.0171
rs12646225	T	0.0251	0.0042	0.0015	0.0175	-0.0162	0.0210
rs13096357	A	0.0236	0.0043	0.0217	0.0194	0.0127	0.0235
rs13157057	T	0.0180	0.0032	-0.0006	0.0155	0.0026	0.0186
rs13253386	T	-0.0201	0.0027	-0.0048	0.0131	0.0011	0.0158
rs13276212	T	0.0151	0.0028	-0.0026	0.0130	-0.0030	0.0157
rs1362739	A	0.0209	0.0027	0.0019	0.0132	0.0081	0.0159
rs1369429	T	0.0176	0.0029	0.0035	0.0135	-0.0017	0.0163
rs144026674	T	0.0413	0.0075	0.0103	0.0415	0.0338	0.0498
rs144246	A	0.0155	0.0028	-0.0477	0.0147	-0.0370	0.0177
rs1455344	A	-0.0162	0.0028	0.0007	0.0129	-0.0008	0.0156
rs146865992	T	0.0475	0.0082	-0.0346	0.0316	-0.0339	0.0387
rs1473474	T	0.0156	0.0027	-0.0325	0.0130	-0.0345	0.0156
rs1589652	A	0.0171	0.0028	-0.0070	0.0130	0.0026	0.0157
rs166820	A	0.0243	0.0036	-0.0023	0.0169	-0.0133	0.0204
rs17106817	T	0.0169	0.0030	0.0170	0.0148	0.0067	0.0178
rs17128425	A	0.0256	0.0045	0.0090	0.0237	0.0077	0.0287

rs176217	T	0.0261	0.0040	-0.0283	0.0219	-0.0202	0.0264
rs17698580	T	0.0190	0.0032	0.0172	0.0153	0.0203	0.0185
rs17826816	A	0.0184	0.0033	0.0135	0.0152	0.0095	0.0184
rs1831539	T	-0.0172	0.0028	-0.0258	0.0130	-0.0237	0.0156
rs1891273	T	0.0154	0.0028	-0.0215	0.0134	-0.0307	0.0162
rs1972860	A	-0.0176	0.0029	0.0154	0.0139	0.0303	0.0168
rs2111490	A	0.0155	0.0028	0.0053	0.0130	0.0084	0.0157
rs2373353	A	-0.0163	0.0029	0.0146	0.0132	0.0064	0.0159
rs2393967	A	-0.0187	0.0030	0.0127	0.0141	0.0101	0.0170
rs2420551	A	-0.0286	0.0043	0.0134	0.0208	0.0061	0.0250
rs2450333	A	-0.0188	0.0028	0.0053	0.0130	0.0083	0.0157
rs2457192	A	-0.0198	0.0031	-0.0054	0.0146	0.0082	0.0175
rs2508713	A	0.0165	0.0028	-0.0088	0.0149	-0.0166	0.0180
rs2558096	T	-0.0156	0.0028	0.0072	0.0132	0.0000	0.0160
rs2576835	A	-0.0194	0.0032	-0.0231	0.0152	-0.0494	0.0183
rs2647995	T	-0.0197	0.0030	-0.0028	0.0142	-0.0088	0.0171
rs2836921	A	0.0203	0.0030	-0.0099	0.0158	-0.0127	0.0190
rs287879	A	-0.0189	0.0031	0.0047	0.0163	0.0259	0.0195
rs2920940	T	-0.0247	0.0033	-0.0141	0.0157	0.0022	0.0190
rs2955280	T	-0.0149	0.0027	0.0127	0.0130	0.0250	0.0156
rs2987390	C	-0.0178	0.0031	0.0038	0.0157	0.0134	0.0189
rs3106666	A	-0.0166	0.0028	0.0275	0.0129	0.0295	0.0156
rs3128341	T	-0.0317	0.0034	0.0029	0.0157	0.0187	0.0189
rs31768	A	0.0182	0.0031	0.0045	0.0137	0.0010	0.0165
rs329672	T	0.0174	0.0029	0.0037	0.0134	-0.0022	0.0162
rs34316	A	0.0210	0.0028	0.0150	0.0131	0.0179	0.0158
rs34320898	C	0.0229	0.0038	0.0100	0.0167	0.0173	0.0202
rs35608616	A	-0.0181	0.0029	0.0020	0.0141	-0.0082	0.0170
rs35760956	A	0.0198	0.0028	-0.0149	0.0139	-0.0174	0.0167
rs36033	T	0.0160	0.0028	0.0292	0.0129	0.0190	0.0156
rs3740422	C	-0.0241	0.0029	-0.0035	0.0136	0.0054	0.0163
rs3749034	A	-0.0193	0.0033	0.0115	0.0150	0.0225	0.0181
rs3823036	T	-0.0190	0.0029	0.0076	0.0138	0.0174	0.0167
rs3843954	C	-0.0208	0.0033	0.0213	0.0147	0.0159	0.0178
rs3860537	T	0.0189	0.0034	0.0057	0.0159	-0.0141	0.0191
rs3896224	A	-0.0153	0.0028	-0.0057	0.0130	-0.0023	0.0156
rs4396807	C	-0.0157	0.0029	0.0193	0.0133	0.0228	0.0161
rs4463213	A	0.0191	0.0027	-0.0158	0.0129	-0.0055	0.0156
rs4484297	C	0.0183	0.0032	-0.0031	0.0140	0.0017	0.0168
rs4731365	A	-0.0192	0.0028	0.0018	0.0130	0.0001	0.0157
rs4793161	A	-0.0177	0.0032	0.0093	0.0150	0.0066	0.0181

rs55754731	T	0.0214	0.0037	0.0021	0.0182	0.0133	0.0220
rs56150095	A	-0.0220	0.0027	-0.0257	0.0129	0.0050	0.0156
rs566237	A	-0.0187	0.0029	0.0034	0.0147	-0.0173	0.0177
rs5753383	A	0.0159	0.0029	0.0307	0.0137	0.0337	0.0165
rs58593843	A	-0.0277	0.0047	0.0268	0.0199	0.0245	0.0240
rs59142272	A	0.0227	0.0037	-0.0049	0.0191	-0.0039	0.0230
rs60262711	T	0.0159	0.0028	0.0035	0.0131	0.0132	0.0158
rs62198803	A	0.0191	0.0032	0.0011	0.0155	-0.0218	0.0186
rs62327949	A	-0.0162	0.0029	0.0255	0.0138	0.0190	0.0166
rs6539284	T	-0.0195	0.0028	0.0073	0.0131	0.0029	0.0158
rs67482514	C	-0.0179	0.0032	-0.0028	0.0147	-0.0220	0.0177
rs6770622	A	-0.0450	0.0069	-0.0381	0.0404	-0.0715	0.0489
rs6819372	A	-0.0198	0.0027	0.0096	0.0130	0.0100	0.0157
rs6840804	A	-0.0166	0.0030	0.0188	0.0138	0.0213	0.0166
rs6860963	T	0.0203	0.0035	-0.0136	0.0152	0.0019	0.0183
rs6903716	A	0.0178	0.0030	0.0059	0.0137	0.0006	0.0165
rs702222	T	-0.0198	0.0029	0.0148	0.0129	0.0264	0.0156
rs7069887	A	0.0225	0.0039	0.0105	0.0199	0.0104	0.0239
rs7116046	T	0.0157	0.0028	-0.0246	0.0131	-0.0198	0.0158
rs71367283	A	0.0560	0.0087	0.0022	0.0468	0.0307	0.0563
rs7172979	T	0.0606	0.0091	0.0268	0.0746	-0.0363	0.0889
rs7248006	T	-0.0192	0.0028	0.0027	0.0130	0.0096	0.0156
rs72739469	T	-0.0344	0.0056	-0.0038	0.0213	0.0080	0.0257
rs73068339	C	0.0189	0.0030	-0.0011	0.0139	0.0025	0.0168
rs7312919	C	0.0181	0.0029	-0.0027	0.0130	0.0005	0.0156
rs73139272	T	-0.0248	0.0041	0.0371	0.0192	0.0592	0.0231
rs7573001	C	-0.0163	0.0029	0.0175	0.0141	0.0221	0.0170
rs75973558	A	0.0256	0.0045	-0.0003	0.0247	-0.0339	0.0295
rs7652296	A	0.0165	0.0028	-0.0103	0.0129	-0.0130	0.0156
rs77418166	T	0.0282	0.0049	-0.0074	0.0267	-0.0436	0.0323
rs7941785	A	0.0155	0.0028	0.0086	0.0136	0.0084	0.0164
rs799444	T	0.0184	0.0028	0.0131	0.0132	0.0070	0.0159
rs8006700	A	-0.0182	0.0029	0.0026	0.0156	0.0196	0.0188
rs8025964	A	0.0170	0.0027	-0.0104	0.0129	-0.0126	0.0156
rs913264	T	0.0197	0.0030	-0.0183	0.0150	-0.0132	0.0181
rs9503599	T	-0.0171	0.0028	0.0430	0.0131	0.0447	0.0158
rs9516855	A	0.0334	0.0061	0.0391	0.0383	0.0048	0.0463
rs9569206	A	-0.0154	0.0028	-0.0008	0.0135	-0.0211	0.0163
rs967569	T	-0.0180	0.0029	0.0203	0.0151	0.0176	0.0182

SNP, single-nucleotide polymorphism; EA, effect allele; SE, standard error; CAD, coronary artery disease; MI, myocardial infarction.

Supplementary Table 4. Statistical power calculation of Mendelian randomization analyses.

Parameter	CAD	MI
Sample size	184,305	171,875
Type-I error rate	0.05	0.05
Proportion of cases	0.33	0.25
Odds ratio of outcome per SD increased intelligence	0.76	0.78
R^2	0.006	0.006
Statistical power	0.98	0.90

SD, standard deviation; R^2, proportion of variance explained for the association between the 121 single nucleotide polymorphisms and the intelligence; CAD, coronary artery disease; MI, myocardial infarction.

Figure S1.

(a)

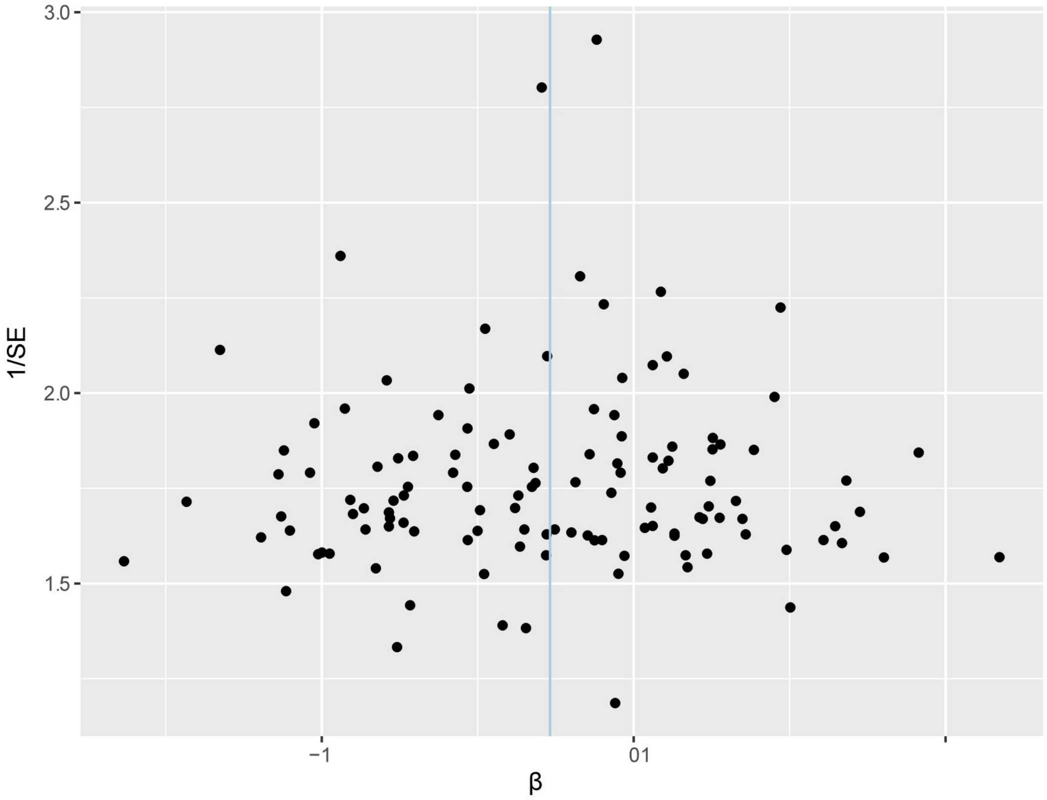

(b)

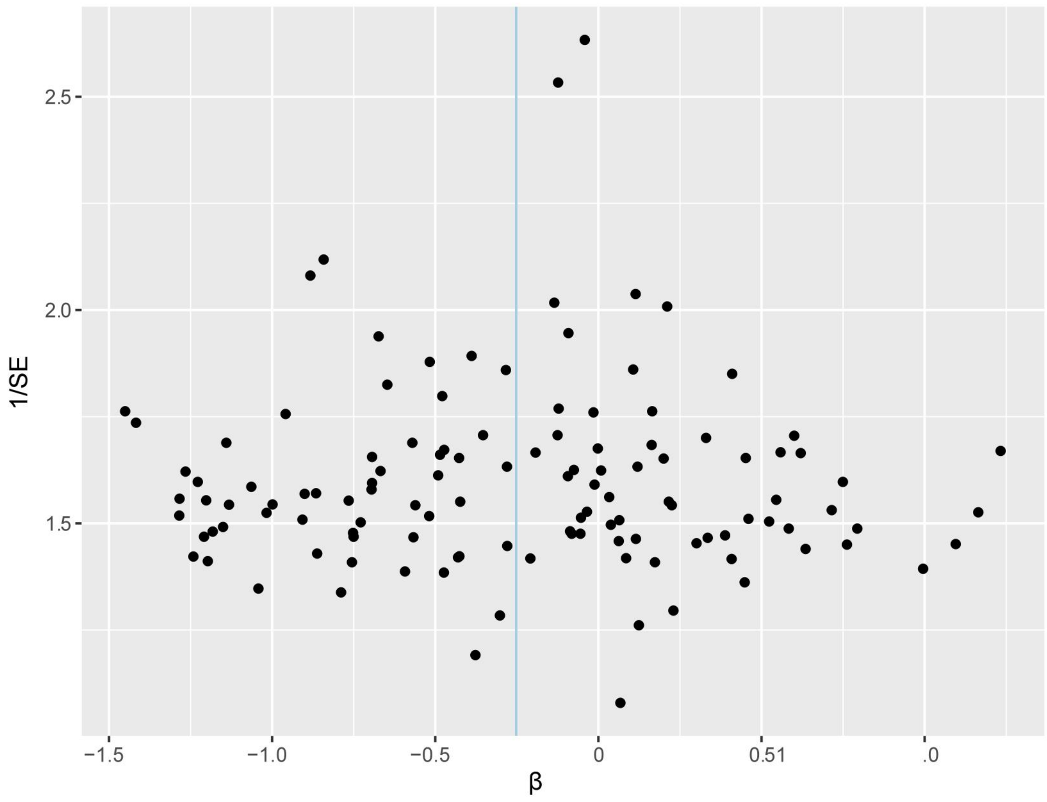

Figure S2.

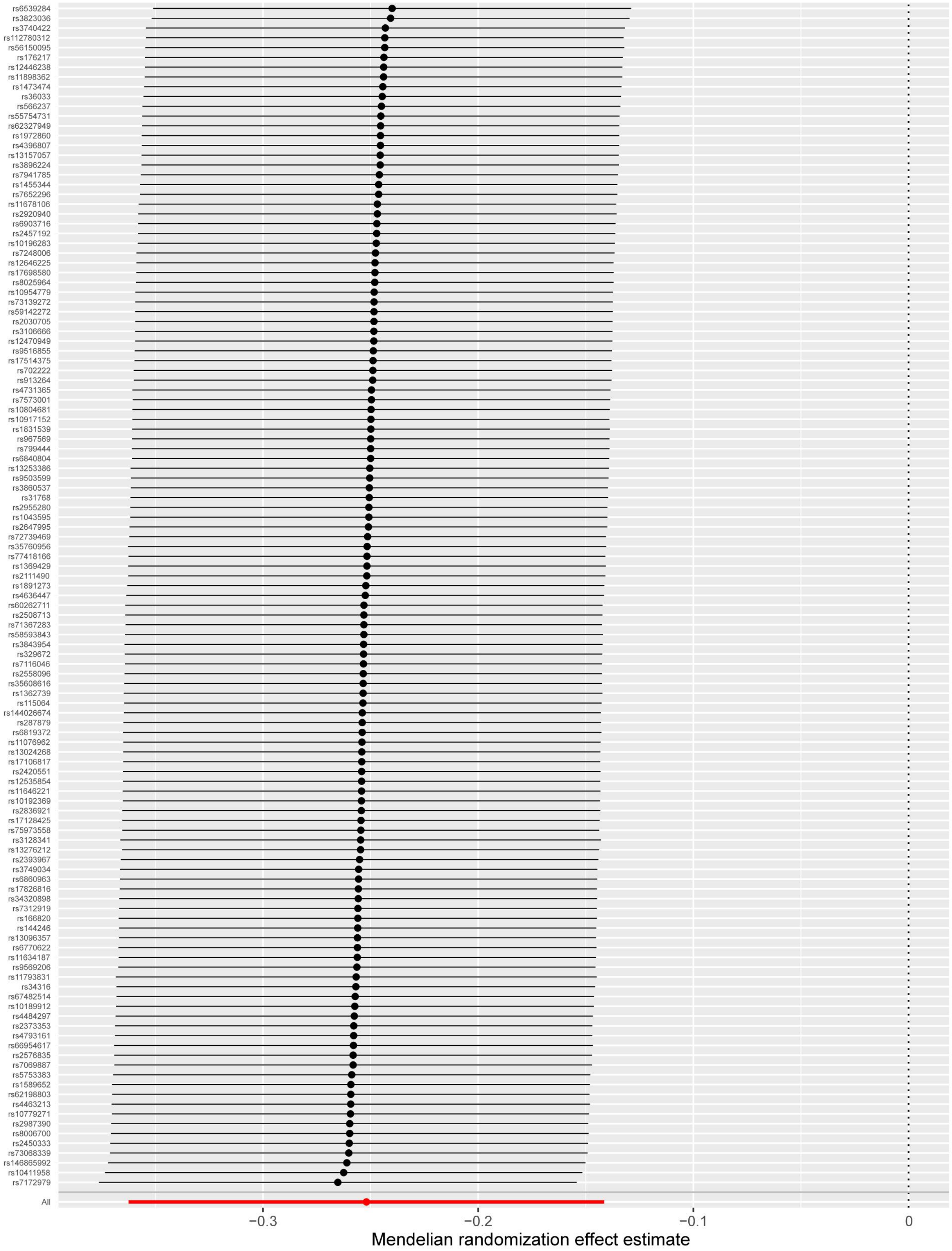

rs6539284
rs3823036
rs3740422
rs112780312
rs56150095
rs176217
rs12446238
rs11898362
rs1473474
rs36033
rs566237
rs55754731
rs62327949
rs1972860
rs4396807
rs13157057
rs3896224
rs7941785
rs1455344
rs7652296
rs11678106
rs2920940
rs6903716
rs2457192
rs10196283
rs7248006
rs12646225
rs17698580
rs8025964
rs10954779
rs73139272
rs59142272
rs2030705
rs3106666
rs12470949
rs9516855
rs17514375
rs702222
rs913264
rs4731365
rs7573001
rs10804681
rs10917152
rs1831539
rs967569
rs799444
rs6840804
rs13253386
rs9503599
rs3860537
rs31768
rs2955280
rs1043595
rs2647995
rs72739469
rs35760956
rs77418166
rs1369429
rs2111490
rs1891273
rs4636447
rs60262711
rs2508713
rs71367283
rs58593843
rs3843954
rs329672
rs7116046
rs2558096
rs35608616
rs1362739
rs115064
rs144026674
rs287879
rs6819372
rs11076962
rs13024268
rs17106817
rs2420551
rs12535854
rs11646221
rs10192369
rs2836921
rs17128425
rs75973558
rs3128341
rs13276212
rs2393967
rs3749034
rs6860963
rs17826816
rs34320898
rs7312919
rs166820
rs144246
rs13096357
rs6770622
rs11634187
rs9569206
rs11793831
rs34316
rs67482514
rs10189912
rs4484297
rs2373353
rs4793161
rs66954617
rs2576835
rs7069887
rs5753383
rs1589652
rs62198803
rs4463213
rs10779271
rs2987390
rs8006700
rs2450333
rs73068339
rs146865992
rs10411958
rs7172979
All
−0.3
−0.2
−0.1
0
Mendelian randomization effect estimate

Figure legends

Supplementary Figure 1. Funnel plot of the Mendelian randomization analyses of genetically predicted intelligence and outcomes. (a) coronary artery disease; (b) myocardial infarction. The dots indicate the causal effect of each SNP. The blue line indicates the averaged effect causal (β) estimate of 121 SNPs using inverse-variance weighted method on X-axis. Y-axis presents the inverse standard error (1/SE) of the causal effect for the SNPs.

Supplementary Figure 2. Leave-one-out analyses of the associations between intelligence and myocardial infarction. The dots indicate the causal effect using inverse-variance weighted method when the SNP is removed. The bars indicate 95% confidence interval.

Code

```
##MRinput
MRInputObject <- mr_input(bx = ,
                          bxse = ,
                          by = ,
                          byse = )

##IVW
IVWObject <- mr_ivw(MRInputObject,
                    model = "default",
                    robust = FALSE,
                    penalized = FALSE,
                    correl = FALSE,
                    weights = "simple",
                    psi = 0,
                    distribution = "normal",
                    alpha = 0.05)

##Weighted Median
WeightedMedianObject <- mr_median(MRInputObject,
                                  weighting = "weighted",
                                  distribution = "normal",
                                  alpha = 0.05,
                                  iterations = 10000,
                                  seed = 314159265)
```

```
##MR-Egger
EggerObject <- mr_egger(MRInputObject,
                        robust = FALSE,
                        penalized = FALSE,
                        correl = FALSE,
                        distribution = "normal",
                        alpha = 0.05)
##maximum likelihood
MaxLikObject <- mr_maxlik(MRInputObject,
                          model = "default",
                          correl = FALSE,
                          psi = 0,
                          distribution = "normal",
                          alpha = 0.05)

##MR-PRESSO
mr_presso(BetaOutcome = "beta.outcome",
                              BetaExposure = "beta.exposure",
                              SdOutcome = "se.outcome",
                              SdExposure = "se.exposure",
                              OUTLIERtest = TRUE,
                              DISTORTIONtest = TRUE,
                              data = dat,
                              NbDistribution = 1000,
                              SignifThreshold = 0.05)
```

```
##MVMR

MRMVInputObject <- mr_mvinput(bx = cbind(GX1, GX2, GX3),
                              bxse = cbind(GX_SE1, GX_SE2, GX_SE3),
                              by = GY,
                              byse = GY_SE)


MRMVObject <- mr_mvivw(MRMVInputObject,
                       model = "default",
                       correl = FALSE,
                       distribution = "normal",
                       alpha = 0.05)
```
